# Phytochemical Prospection and Modulation of Antibiotic Activity In Vitro by *Lippia origanoides* H.B.K. in Methicillin Resistant *Staphylococcus aureus*

**DOI:** 10.1155/2014/305610

**Published:** 2014-02-10

**Authors:** Humberto Medeiros Barreto, Filipe Cerqueira Fontinele, Aldeídia Pereira de Oliveira, Daniel Dias Rufino Arcanjo, Bernadete Helena Cavalcanti dos Santos, Aislan Pereira Lira de Abreu, Henrique Douglas Melo Coutinho, Romezio Alves Carvalho da Silva, Taciana Oliveira de Sousa, Maria das Graças Freire de Medeiros, Antonia Maria das Graças Lopes Citó, José Arimateia Dantas Lopes

**Affiliations:** ^1^Laboratory of Microbiology and Immunology, Campus Amílcar Ferreira Sobral, Universidade Federal do Piauí, BR 343, Km 3,5 Bairro Meladão, 64800-000 Floriano, PI, Brazil; ^2^Center for Research on Medicinal Plants, Universidade Federal do Piauí, Bairro Ininga, 64049-550 Teresina, PI, Brazil; ^3^Laboratory of Clinical Microbiology, Universidade Federal da Paraiba, Cidade Universitária, 58051-900 João Pessoa, PB, Brazil; ^4^Laboratory of Microbiology, Faculdade de Ensino Superior de Floriano, Bairro Manguinha, 64800-000 Floriano, PI, Brazil; ^5^Laboratory of Microbiology and Molecular Biology, Regional University of Cariri, Bairro Pimenta, 63105-100 Crato, CE, Brazil; ^6^Laboratory of Natural Products, Universidade Federal do Piauí, Bairro Ininga, 64049-550 Teresina, PI, Brazil

## Abstract

The *Lippia origanoides* H.B.K. ethanol extract (LOEE) and hexane (LOHEX), dichloromethane (LODCM), and ethyl acetate (LOEA) fractions were tested for their antimicrobial activity alone or in combination with antibiotics against a methicillin resistant *Staphylococcus aureus* (MRSA) strain. The natural products did not show antimicrobial activity against multidrug resistant strain at the clinically significant concentrations tested. However, a modulatory effect in the antibacterial activity of the neomycin and amikacin was verified when LOEE, LOHEX and LODCM were added to the growth medium at subinhibitory concentrations. A similar modulation was found when the natural products were changed for chlorpromazine, an inhibitor of bacterial efflux pumps, suggesting the involvement of resistance mediated by efflux system in the MRSA tested. The fractions LOHEX and LODCM showed a modulatory activity bigger than their majority compounds (carvacrol, thymol, and naringenin), indicating that this activity is not due to their majority compounds only, but it is probably due to a synergism between their chemical components. These results indicate that *L. origanoides* H.B.K. can be a source of phytochemicals able to modify the phenotype of resistance to aminoglycosides in MRSA.

## 1. Introduction

Methicillin resistant *S. aureus *(MRSA) remains as an important cause of infectious diseases acquired in hospitals and communities worldwide [1–3]. This pathogen is a common cause of cutaneous and soft tissue infections, as well as invasive illness, such as bacteremia, septic arthritis, osteomyelitis and necrotizing pneumonia [4, 5]. The therapy of these infections is often complicated once many MRSA strains carry multiple genes of antibiotic resistance [6, 7].

The high prevalence of infectious diseases caused by MRSA and other multidrug resistant bacteria has motivated the search for new antimicrobial agents and/or new compounds able to potentiate the antimicrobial activity of old antibiotics [8–10]. Several studies have evidenced that secondary metabolites derived from plant metabolism may be active against multi-drug resistant bacteria, including MRSA [11, 12].

**Figure 1:**
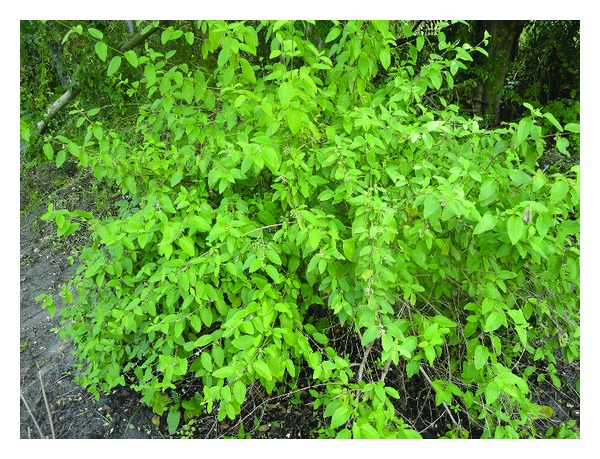
*Lippia origanoides* H.B.K.

The family Verbenaceae is made up of 75 genera with about 3000 species and the genus *Lippia* has about 200 species distributed in countries of Central and South America, as well as countries of tropical Africa [13]. *Lippia origanoides *H.B.K. (Figure 1) is a medicinal plant known in the Piauí state as *alecrim-do-campo*. Its leaves have been used as culinary seasoning and in traditional medicine as remedy for gastrointestinal disorders and as a general antiseptic for mouth, throat, and wounds [14, 15]. The essential oil is the most studied product obtained from *L. origanoides *H.B.K., which exhibits antigenotoxic effect in bacterial cells, antioxidant activity, low toxicity, as well as antimicrobial and antiparasite activities [16–21].

On the other hand, biological activities of extracts and partition fractions obtained from *L. origanoides *H.B.K need further studies, and as far as we know, the antibiotic-resistance modifying activity of its extracts has not been investigated yet. In the present study, the ethanol extract obtained from *L. origanoides *H.B.K., and different partition fractions and their majority compounds were tested for their antimicrobial activity alone or in combination with aminoglycoside antibiotics, aiming to evaluate its potential as a natural source of secondary metabolites acting as modulators of antibiotic resistance.

## 2. Materials and Methods

### 2.1. Strains and Drugs

All tests were performed with multidrug resistant bacterial strain of *Staphylococcus aureus *SA10 isolated from rectal swab (resistant to cephalothin, cephalexin, cefadroxil, penicillin G, ampicillin, amoxicillin, oxacillin, erythromycin, clarithromycin, azithromycin, clindamycin, moxifloxacin, ciprofloxacin, levofloxacin, nalidixic acid, tetracycline, neomycin, gentamicin and amikacin), which was kindly provided by Laboratory of Clinical Microbiology of the Universidade Federal da Paraiba. The standard strain *Staphylococcus aureus *ATCC25923 was used as positive controls. The strains were maintained on Nutrient Agar (Himedia, India) slant at 4°C, and prior to assay the cells were grown overnight at 37°C in Brain Heart Infusion (BHI, Himedia, India). Neomycin, amikacin, naringenin, carvacrol, thymol, and chlorpromazine were obtained from Sigma Chemical Corp., St. Louis, MO, USA. Antibiotics and chlorpromazine were dissolved in sterile water. Naringenin, carvacrol, and thymol were dissolved in dimethylsulfoxide (DMSO-MERCK) and the stock solutions were diluted with sterile water.

### 2.2. Plant Material

Leaves of *Lippia origanoides *H.B.K. were collected in the county of José de Freitas (latitude 04°45′23′′ south and longitude 42°34′32′′ west), Piauí, Brazil. The plant material was identified and a voucher specimen was deposited with the number TEPB09205 at the Herbarium ‘‘Graziela Barroso” of Universidade Federal do Piauí (UFPI).

### 2.3. Preparation of Extracts and Fractions

The leaves were dried at room temperature and powdered. The powdered material (1,120 g) was extracted by maceration using ethanol as solvent in the ratio of 1 : 3 (m/v) and the homogenate was allowed to stand for 72 h at room temperature. This procedure was realized in triplicate. The supernatants were then filtered, gathered, and concentrated under vacuum in a rotary evaporator (model Q-344B-Quimis, Brazil) and ultra thermal bath (model Q-214M2-Quimis, Brazil), yielding 309.5 g of ethanol extract (LOEE). A part of this extract was suspended in ethanol/water (1 : 1, v/v) and partitioned in solvents with increasing polarity (hexane, dichloromethane, and ethyl acetate), obtaining fractions hexane (LOHEX), dichloromethane (LODCM), and ethyl acetate (LOEA), respectively. The fractions were concentrated under vacuum in a rotary evaporator and lyophilized. After extraction with ethanol, the residue was macerated with ethanol/water (1 : 1, v/v) in the ratio of 1 : 3 (m/v), and the homogenate was allowed to stand for 72 h at room temperature. This procedure was repeated for three consecutive times. The supernatants were then filtered, gathered and concentrated under vacuum in a rotary evaporator and lyophilized, thereby obtaining the hydro alcoholic extract (LOHA).

### 2.4. Gas Chromatography/Mass Spectrometry (GC-MS)

The fractions' constituents were converted to silylated derivatives according to Isidorov et al. [22], with modifications. Two milligrams of each fraction were mixed with 100 *μ*L of bis(trimethylsilyl)trifluoroacetamide and trimethylsilyl chloride 1%. The mix was maintained at 85°C under agitation for 1 hour. The silylated fractions were analyzed after injection of 1 µL in a gas chromatograph (Shimadzu GC-17A) with a flame ionization detector (FID) model ISQ and coupled to an mass spectrometer model GCMS-QP5050A equipped with a DB-5 HT (Agilent, Palo Alto, CA, USA) 95% methyl-polysiloxane and 5% phenyl capillary column (intern diameter = 0.25 mm, length 30 m, film thickness = 0,1 µm). Operating conditions were as follows: injector temperature, 260°C; detector temperature, 300°C; carrier gas (Helium), flow rate of 1 mL min^−1^. Oven temperature was initially 60°C (0,5 min) and was then raised to 260°C (5 min) at a rate of 6°C min^−1^; then it was heated to 300°C (10 min) at a rate of 12°C min^−1^. The mass spectrometry conditions were as follows: scan mode with acquisition time of 52.21 min; ionization voltage, 70 eV; mass range, 40–650 Da; ion source temperature, 200°C. Compounds were preliminarily identified by characteristic fragmentation and by comparison of NIST 2.0 mass spectra libraries.

### 2.5. Evaluation of the Antibacterial Activity

Stock solutions of LOHEX, LODCM, and LOEA were prepared by dissolving 10 mg of each product in 1 mL of dimethyl sulfide, thus starting with an initial concentration of 10 mg/mL. The resulting solution was then diluted to 1024 *μ*g/mL in sterile water. Minimal inhibitory concentrations (MICs) of the LOHEX, LODCM, LOEA, carvacrol, thymol, naringenin, and chlorpromazine were determined by the microdilution assay in BHI broth 10% with bacterial suspensions of 10^5^ CFU/mL. The final concentrations of the fractions and compounds ranged from 512 to 8 *μ*g/mL [23]. MICs of neomycin and amikacin were determined for the same method with antibiotic concentrations ranging from 2,500 to 2.4 *μ*g/mL.

### 2.6. Modulation of the Antibiotic Activity

For evaluation of extracts and their majority compounds as antibiotic resistance modulators, MICs of the antibiotics were determined in the presence or absence of subinhibitory concentrations (1/8 MIC) of the extracts (128 *μ*g/mL), naringenin (128 *μ*g/mL), carvacrol and thymol (32 *μ*g/mL), and chlorpromazine (8 *μ*g/mL) [24]. The plates were incubated at 37°C for 24 h.

### 2.7. Statistical Analysis

Each experiment was performed six times and the results were normalized by calculation of geometric mean values. Error deviation and standard deviation of the geometric mean were revealed. Statistical analyses were performed using GraphPad Prism, version 5.02. Differences between treatment with antibiotics in the absence and the presence of the *L. origanoides *extract and fractions, as well as carvacrol, thymol, naringenin, and chlorpromazine were examined using two-way analysis of variance (ANOVA). The differences mentioned above were analyzed by Bonferroni posttest and they were considered statistically significant when *P* < 0.05.

## 3. Results and Discussion

### 3.1. Evaluation of the Antibacterial Activity

The antibacterial activity of the natural products from *L. origanoides *H.B.K., as well as the majority compounds of the LOHEX (carvacrol and thymol) and LODCM (naringenin) was tested by microdilution method against *S. aureus *strains (ATCC 25923 and SA10). Plant extracts are considered as having a good inhibitory activity if they present MICs ≤ 100 *μ*g/mL, a moderate inhibitory activity if they present MICs ranging from 100 to 500 *μ*g/mL, a weak inhibitory activity if they present MICs ranging from 500 to 1000 *μ*g/mL, and no inhibitory activity if they present MICs > 1000 *μ*g/mL [25, 26]. According to these criteria, the extract and fractions, as well as, naringenin did not show antibacterial activity against the *S. aureus* strains (once they showed MICs ≥ 1024 *μ*g/mL). On the other hand, carvacrol and thymol presented a moderate inhibitory activity (MIC 256 *μ*g/mL) against the *S. aureus* strains.

### 3.2. Modulation of Antibiotic Activity

**Figure 2:**
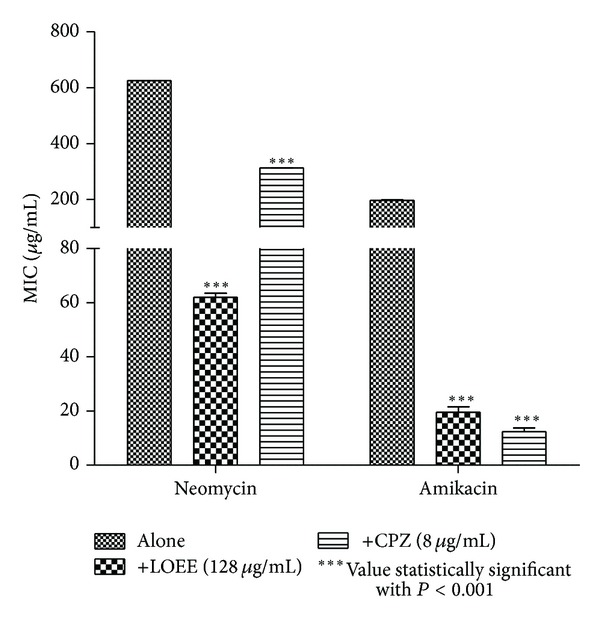
MIC of the antibiotics in the absence and presence of LOEE and chlorpromazine (CPZ) at subinhibitory concentrations for *S. aureus* strain SA10.

**Figure 3:**
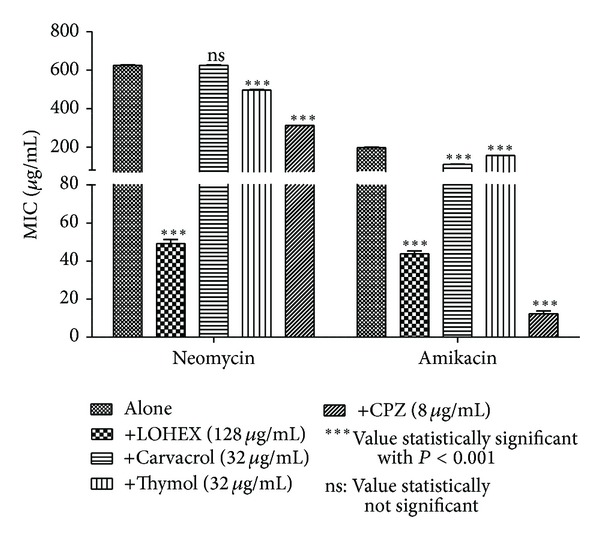
MIC of the antibiotics in the absence and presence of LOHEX, carvacrol, thymol, and chlorpromazine (CPZ) at subinhibitory concentrations for *S. aureus* strain SA10.

**Figure 4:**
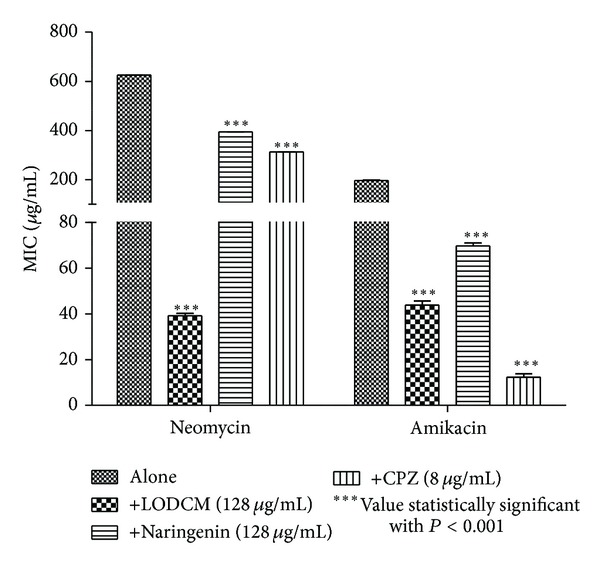
MIC of antibiotics in the absence and presence of LODCM, naringenin, and chlorpromazine (CPZ) at subinhibitory concentrations for *S. aureus* strain SA10.

When LOEE or the fractions LOHEX and LODCM were added to the growth medium at subinhibitory concentration, a reduction in the MIC of at least 10-fold for neomycin and at least 5-fold for amikacin was verified (Figures 2–4). To the best of our knowledge, this is the first time that *Lippia origanoides* H.B.K. extracts are described as enhancers of the aminoglycosides activity against MRSA, and the results were compatible with those found for other medicinal plants [10, 27–29].

After crossing the cell wall and the cell membrane, aminoglycosides bind to the ribosomal subunit 30S, interfering with the binding of mRNA, leading to the synthesis of anomalous proteins, which are inserted into plasma membrane changing its permeability [30]. Resistance to aminoglycosides in *S. aureus* is frequently related to enzymatic inactivation [31], but it can be mediated by membrane transporter proteins able to pump the antibiotic to the extracellular medium, as LmrS efflux protein [32, 33].

Decrease in the MICs of antibiotics tested was also verified when LOEE, LOHEX, and LODCM were replaced by chlorpromazine at subinhibitory concentration. The role of chlorpromazine as inhibitor of bacterial efflux systems has already been well studied [34, 35]. This result indicates a possible mechanism mediated by efflux pump for resistance to neomycin and amikacin in the SA10 strain, which could be inhibited by phytochemicals present in LOEE and in both LOHEX and LODCM fractions, although further studies are needed to confirm such mechanism.

### 3.3. Phytochemical Prospection

Through phytochemical prospecting of the fractions studied, several classes of secondary metabolites were identified. The majority compounds found in the LOHEX were the monoterpenoids carvacrol and thymol, besides other minority components (Table 1 and Figure 6). The antimicrobial activity of phenolic compounds such as carvacrol and thymol has been demonstrated previously [36], including against MRSA [37]. Due to their lipophilicity, these compounds can intercalate in the phospholipid bilayer, increasing the permeability of the cell membrane, dissipation of the proton-motive force, and leakage of inorganic ions, resulting in lysis and cell death [38, 39].

Flavonoids aglycones were the main phytochemicals found in the fraction LODCM (Table 2 and Figure 6), including the flavanones naringenin (in the keto and enol forms), eriodictyol and hesperetin, the isoflavones dihydroglycitein and 3′hydroxy-irilone, and the flavan-3-ol (+)catechin. Some lipophilic flavonoids can react with bacterial membrane [40], besides forming complexes with proteins [41]. As already mentioned, these mechanisms may increase the membrane permeability and inhibit efflux systems dependent of proton-motive force, leading to a bigger accumulate of antibiotics into the bacterial cell [34]. In fact, the modulatory effect of flavonoids on resistance to quinolones and tetracycline has already been verified in *Staphylococcus aureus* [42–44].

With regard to the modulatory effect found in this study, it could be attributed to an interaction of LOHEX and LODCM components in the plasma membrane, leading to an increase in the cell permeability to the aminoglycosides. Furthermore, this interaction could inhibit efflux systems dependent of proton-motive force, as LmrS protein, contributing to the increase in the intracellular levels of antibiotics and leading to an enhancement of the aminoglycoside activity against MRSA.

Thymol and carvacrol did not modulate the antistaphylococcal activity of the neomycin or amikacin in the SA10 strain (Figure 3), and the modulatory activity verified for naringenin was lower than that showed by LODCM (Figure 4). These results suggest that the modulatory activity verified for LOHEX and LODCM is not only caused by their majority compounds, but it could be due to a synergism among their components. However, further investigations with different phytochemicals alone and in different combinations between them are needed to test this hypothesis.

**Figure 5:**
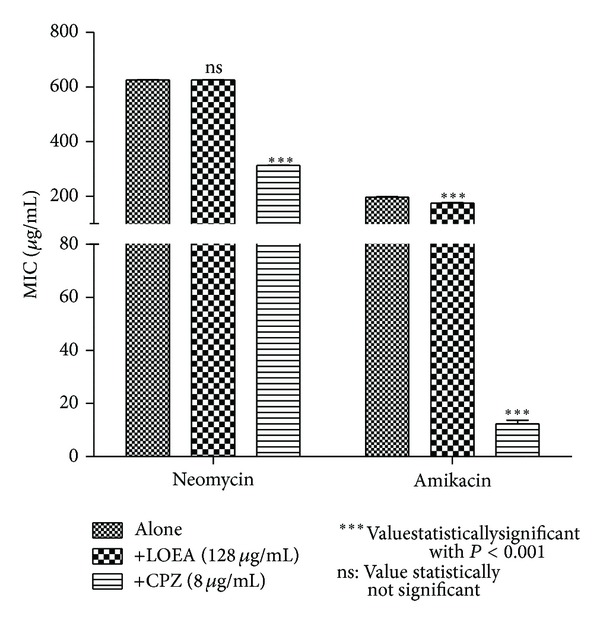
MIC of antibiotics in the absence and presence of LOEA and chlorpromazine (CPZ) at subinhibitory concentrations for *S. aureus* strain SA10.

**Figure 6:**
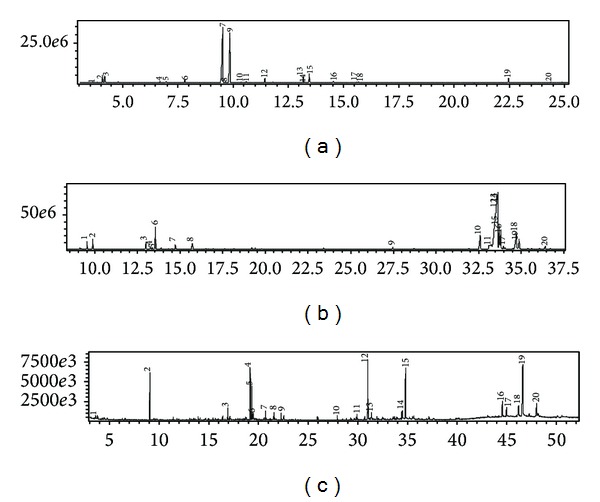
Total ions chromatogram obtained from LOHEX (a), LODCM (b), and LOEA (c) fractions by GC-MS analysis.

LOEA also did not modulate the activity of the aminoglycosides tested (Figure 5). In this fraction was found a high content of irilone *O-*heterosides, besides the aglycone catechin (Table 3 and Figure 6). It has been demonstrated that addition of hydrophilic groups as glycosides becomes the compound less effective at inhibiting Gram-positive bacteria [45, 46]. It was suggested that the lack of affinity for the phospholipid bilayer or specific receptors on the cell membrane is the motive for this decrease in the antibacterial activity [47]. The lack of modulatory effect in the activity of the aminoglycosides tested showed by LOEA also may be related to the bigger hydrophilicity of the compounds present in this fraction.

## 4. Conclusions

In the present work, it was verified that LOEE and the nonpolar fractions LOHEX and LODCM were able to potentiate the activity of neomycin and amikacin against a MRSA strain in vitro, which may be related to the low polarity of its phytochemicals. Aminoglycosides have been used in the initial empiric treatment of endocarditis caused by Gram-positive bacteria, including *S. aureus*, but the high frequency of resistant strains and adverse reactions have discouraged its clinical use [48]. The results obtained indicated that *Lippia origanoides *H.B.K. could be a source of secondary metabolites for use in association with neomycin and amikacin in the antibiotic chemotherapy of infections caused by MRSA.

## Figures and Tables

**Table 1:** Chemical composition of *Lippia origanoides *H.B.K. hexane fraction (LOHEX).

Peaks	Compound	RT^a^	Area (%)
1	Ethylene glycol	3.600	0.45
2	*p*-Cymene	4.054	2.44
3	1.8-Cineol	4.161	3.74
4	Terpinen-4-ol	6.676	0.51
5	*α*-Terpineol	6.944	0.87
6	Thymol methyl ether	7.799	1.32
7	Thymol	9.514	38.67
8	*α*-Terpineol	9.636	0.33
9	Carvacrol	9.855	36.17
10	Not identified	10.358	0.42
11	*α*-Copaene	10.600	0.61
12	*β*-Caryophyllene	11.439	2.01
13	Theophylline	13.176	2.72
14	*β*-Bisabolene	13.244	0.50
15	Not identified	13.458	4.79
16	Caryophyllene oxide	14.553	0.95
17	Not identified	15.623	0.38
18	Not identified	15.684	0.37
19	Palmitic acid	22.518	2.44
20	Menthol	24.449	0.31

^a^Retention time.

**Table 2:** Chemical composition of *Lippia origanoides *H.B.K. dichloromethane fraction (LODCM).

Peaks	Compound	RT^a^	Area (%)
1	Thymol	9.524	1.26
2	Carvacrol	9.864	1.77
3	2,3-Dimethylcyclopentenol	12.983	1.41
4	Theophylline	13.239	0.48
5	Cycloleucine	13.357	0.34
6	Not identified	13.547	4.98
7	7-Methylxanthine	14.700	0.64
8	Not identified	15.713	1.37
9	3′-Hydroxy-irilone	27.474	0.48
10	Dihydroglycitein	32.615	4.77
11	Not identified	33.277	4.49
12	Not identified	33.550	29.49
13	Naringenin (enol form)	33.593	15.56
14	Naringenin (keto form)	33.637	10.4
15	Not identified	33.754	6.3
16	Eriodictyol	33.830	4.49
17	Hesperetin	33.976	0.54
18	Not identified	34.755	7.82
19	(+)Catechin	34.899	2.62
20	2,4,6-Trihydroxybenzoic acid	36.439	0.79

^a^Retention time.

**Table 3:** Chemical composition of *Lippia origanoides *H.B.K. ethyl acetate fraction (LODCM).

Peaks	Compound	RT^a^	Area (%)
1	Ethylene glycol	3.625	0.97
2	Glycerol	9.080	8.19
3	Not identified	16.937	2.22
4	Protocatechuic acid	19.191	9.68
5	L-Rhamnose	19.366	5.63
6	2-Keto-D-gluconic	19.492	1.15
7	D-Xylopyranose	20.740	2.12
8	Gallic acid	21.575	1.46
9	D-Mannose	22.312	1.27
10	Not identified	27.956	0.92
11	Not identified	29.948	1.06
12	Isomaltulose	31.022	16.37
13	Isomaltulose	31.377	1.29
14	D-Turanose	34.476	1.62
15	Catechin	34.838	11.61
16	3′-Hydroxy-5-furanoside-dihydroirilone	44.546	4.54
17	3′-Hydroxy-5-inosose-irilone	44.980	2.32
18	3′-Hydroxy-5-piranoside-dihidroirilone	46.179	3.07
19	3′-Hydroxy-5-inosose-dihydroirilone	47.991	5.08

^a^Retention time.
